# 20 years of the Health and Environmental Surveillance Secretariat: an analysis of two decades and perspectives

**DOI:** 10.1590/S2237-962220230004000016.EN

**Published:** 2023-12-04

**Authors:** Ethel Leonor Noia Maciel

**Affiliations:** 1 Ministério da Saúde, Secretaria de Vigilância em Saúde e Ambiente, Brasília, DF, Brazil

When Silva Junior published the article *A nova face da vigilância epidemiológica* (The new face of epidemiological surveillance) two decades ago,^1^ presenting the new structure of the Ministry of Health with the creation of the Health Surveillance Secretariat (Secretaria de Vigilância em Saúde - SVS), I was a doctoral student in epidemiology, and I vividly recall the collective satisfaction that accompanied that announcement and the promise of a new era at the beginning of the government in 2003.

The concept of bringing together, under the same secretariat, programs and control of communicable diseases, which were previously scattered, along with chronic diseases and non-communicable diseases, ensuring their more effective monitoring, invigorated the entire public health area.

Over two decades, there have been many significant changes in the actions of the SVS. Notably, it played a coordinating role in implementing the National Health Surveillance Policy (Política Nacional de Vigilância em Saúde - PNVS),^2^ established in 2018, and the National Occupational Health Policy,^3^ created in 2012, which encompasses the National Network for Comprehensive Occupational Health Care (Rede Nacional de Atenção Integral à Saúde do Trabalhador - RENAST), comprising 215 occupational health reference centers (centros de referência em saúde do trabalhador - CERESTs), across several states and municipalities in Brazil.

A milestone was reached in 2023 with the recognition of endemic disease control agents (agentes de combate às endemias - ACEs) as health professionals who perform surveillance, disease prevention and control, and health promotion activities.^4^ Through its Department of Strategic Health Surveillance Articulation (Departamento de Articulação Estratégica em Vigilância em Saúde - DAEVS), the Health and Environmental Surveillance Secretariat (Secretaria de Vigilância em Saúde e Ambiente - SVSA) monthly allocates resources to Brazilian states, the Federal District and municipalities to support health and environmental surveillance actions. This is accomplished through Financial Incentives (FI) for ACEs and the Fixed Health Surveillance Floor (Piso Fixo de Vigilância em Saúde - PFVS), among other mechanisms.

Through the Department of Communicable Diseases (Departamento de Doenças Transmissíveis - DEDT), the Department of Epidemiological Analysis and Surveillance of Non-Communicable Diseases (Departamento de Análise Epidemiológica e Vigilância de Doenças Não Transmissíveis - DAENT) and the Department of HIV/AIDS, Tuberculosis, Viral Hepatitis and Sexually Transmitted Infections (Departamento de HIV/AIDS, Tuberculose, Hepatites Virais e Infecções Sexualmente Transmissíveis - DATHI), the SVSA promotes the improvement and broadening of programs and actions for the surveillance, control and elimination of communicable diseases, as well as the surveillance and monitoring of non-communicable diseases, accidents and violence nationwide.

It is worth highlighting the role of the Department of Public Health Emergencies (Departamento de Emergências em Saúde Pública - DEMSP), in the coordination of the National Network for Surveillance, Alert and Response to Public Health Emergencies, comprising 190 units of the Center for Strategic Information in Health Surveillance (Centro de Informações Estratégicas em Vigilância em Saúde - CIEVS); the National Hospital Epidemiological Surveillance Network (Rede Nacional de Vigilância Epidemiológica Hospitalar - RENAVEH); the National Program for Health Surveillance of Risks Associated with Disasters (Programa Nacional de Vigilância em Saúde dos Riscos Associados aos Desastres - VIGIDESASTRES); and the Field Epidemiology Training Program Applied to the Services of the Brazilian National Health System (Programa de Treinamento em Epidemiologia de Campo Aplicado aos Serviços do Sistema Único de Saúde - EPISUS). In addition to these Secretariat structures, there is the coordination of the National System of Public Health Laboratories in Brazil and the National Immunization Program, responsible for one of Brazil’s most successful public policies, which this year celebrated its 50^th^ anniversary.

Furthermore, the establishment of the SVS encompassed the recognition of the need to invest in a scientific journal with the mission of disseminating epidemiological knowledge applicable to surveillance, prevention and control of diseases and health conditions of public health interest within the scope of the SUS. The former SUS Epidemiological Report (*Informe Epidemiológico do SUS*), created in 1992, underwent restructuring to meet these needs, giving rise to Epidemiology and Health Services: journal of the Brazilian National Health System (*Epidemiologia e Serviços de Saúde: revista do SUS - RESS*). Beyond its primary mission, the journal has also contributed to the consolidation of numerous postgraduate programs in the public health area, especially those with a professional profile. Currently, our journal is classified in stratum A3 by the Coordination for the Improvement of Higher Education Personnel (Coordenação de Aperfeiçoamento de Pessoal de Nível Superior - CAPES) and its excellence is recognized nationally and internationally. Over two decades, the journal has modernized and indexed in major national databases (SciELO) and international ones (Scopus, Medline, Web of Science and Pubmed Central), regularly publishing articles in both Portuguese and English versions, boasting an editorial board featuring renowned epidemiologists and public health experts.

This year, as the former SVS celebrates its 20^th^ anniversary, new winds are blowing. For the first time, we have a woman leading the Ministry of Health, researcher Nísia Trindade Lima.^5^ Currently, SVS takes on a new scope with the inclusion of the term “environment”,^6^ thus recognizing the inseparable relationships among human health, animal health and the environment, which is both determinant and determined by health.

The new SVSA, therefore, acknowledges the central role of climate change^7^ in the emergence of diseases and the importance of environmental health in this context. Aligned with occupational health, they are part of the guiding principle for healthy populations, with the Department of Environmental and Occupational Health Surveillance (Departamento de Vigilância em Saúde Ambiental e Saúde do Trabalhador - DSAST) serving as the organizational structure catalyst for these actions. The SVSA also recognizes the uniqueness of how accidents and violence impact population health and, thus, creates, in 2023, the coordination of accidents and violence within the DAENT, moving away from the generic term “health conditions”. The creation of the National Center for Epidemiological Intelligence (Centro Nacional de Inteligência Epidemiológica - CNIE), scheduled for 2024, is a cornerstone for ensuring greater capacity to use data to generate timely and qualified information. Alongside the transformation of the National Immunization Program (Programa Nacional de Imunizações - PNI) into a department (DPNI), with administrative arrangements aimed at enhancing program management, these initiatives are examples of this new era.

The elevation of laboratory-based health surveillance to a national priority in the new Growth Acceleration Program (Plano de Aceleração do Crescimento - PAC), unequivocally demonstrates the importance of laboratory diagnostics in preparing for future health emergencies. The incorporation of the Instituto Evandro Chagas and the Centro Nacional de Primatas into the SVSA enhances laboratory-based health surveillance in preparation and response to public health emergencies, positioning the North region as a leader in health actions in and for Brazil.

After a pandemic period, during which science denial was the normative conduct of management, repositioning science at the center of decision-making is not only symbolic, but also essential.

The SVSA, formerly SVS, has grown, flourished, faced threats of suffocation, but has resisted and currently, under the leadership of a minister who understands and advocates for the SUS, stands ready for the challenges of the coming decades.
